# Explaining and predicting consumer decision-making in E-commerce using an intelligent hybrid framework for impulsive and planned purchase behavior

**DOI:** 10.1038/s41598-026-47284-1

**Published:** 2026-04-11

**Authors:** Na Cui

**Affiliations:** 1 School of Supply Chain Management, NINGBO POLYTECHNIC UNIVERSITY, Ningbo, 315800 Zhejiang China; 2 Port and Shipping Digital Supply Chain Research Institute, Ningbo, 315800 Zhejiang China

**Keywords:** E-commerce analytics, Hyperparameter tuning, Impulse buying prediction, Metaheuristic algorithm, Hybrid machine learning, Dynamic pricing, Engineering, Mathematics and computing

## Abstract

Accurately distinguishing between impulsive and planned purchasing behavior remains a critical challenge in e-commerce analytics, given the multidimensional and dynamic nature of consumer data. Existing machine learning approaches often rely on conventional hyperparameter tuning strategies, which may limit model robustness and generalization. This study proposes a hybrid framework that integrates two bio-inspired metaheuristic algorithms—Walrus Optimization Algorithm (WaOA) and Weevil Damage Optimization Algorithm (WDOA)—with three gradient boosting classifiers (XGBoost, LightGBM, and CatBoost) to improve hyperparameter optimization and classification performance. Empirical evaluation indicates that the WaOA-optimized XGBoost model achieved strong predictive performance, reaching F1-scores of 0.9879 for impulsive purchases and 0.9798 for need-based purchases, with an overall testing accuracy of 97.3%. Although statistical tests suggest that performance differences among the evaluated models are not significant, the optimized configurations demonstrate consistently high predictive capability across evaluation metrics. Feature importance analysis identifies product rating, customer satisfaction, and loyalty program membership as key predictors of consumer decision type. The proposed framework demonstrates promising performance and interpretability, offering practical support for data-driven decision-making in digital retail environments.

## Introduction

Consumer behavior has become more intricate and varied as competition in the global marketplace has intensified. This complexity is often beyond the scope of traditional marketing paradigms, which primarily rely on static segmentation and aggregate historical data^1,2^. For instance, the traditional 4P framework, which consists of product, price, promotion, and place, was developed during a time when information flows were constrained, and consumer segments were comparatively uniform^3^. In contrast, modern consumers use mobile applications, social media, and online transactions to generate large, fast-moving data streams. Static marketing models cannot readily account for moment-to-moment decision-making, personalized motivations, and nuanced preferences captured by these real-time data flows. As a result, businesses must quickly embrace more flexible, data-driven methods to find new client segments, improve retention tactics, and increase market share^4^. A combination of cultural, social, and psychological factors influences consumer behavior. Baseline preferences are shaped by cultural norms and societal influences, but additional variability is introduced by personal cognitive biases and emotional states.

Additionally, consumers must sift through vast amounts of content before making purchasing decisions due to the information overload caused by the widespread use of digital channels. Consumer demands have changed in this context, becoming more contextually dependent, flexible, and highly personalized^5^. An individual’s preference for a brand, for example, may depend on peer endorsements, in-the-moment promotional stimuli, and latent lifestyle aspirations in addition to product specifications. When faced with such heterogeneity, traditional marketing strategies that rely on broad demographic segments often fail, which emphasizes the need for new analytical frameworks that can handle multidimensional, high-frequency data^6,7^.

One notable solution to these constraints is precision marketing^8^. The transition from mass marketing to precision-driven strategies has been accelerated by the availability of granular behavioral data generated through clickstreams, browsing histories, transaction logs, and social media interactions^9^. Rather than relying on broad demographic segmentation, precision marketing leverages real-time behavioral signals to infer consumer intent and deliver context-aware interventions^10,11^. However, effectively operationalizing personalization requires predictive models capable of handling high-dimensional, heterogeneous, and rapidly evolving datasets. Traditional regression-based methods often struggle with non-linearity and scalability, while standard machine learning approaches may suffer from unstable hyperparameter selection and limited generalization^12^. Advanced ensemble techniques, particularly gradient boosting frameworks, offer strong predictive capacity, but their performance is highly sensitive to hyperparameter configuration. Integrating optimized machine learning pipelines with behaviorally grounded features enables not only improved classification accuracy but also more reliable support for applications such as dynamic pricing, inventory planning, and customer retention strategies^13^.

Despite significant advances in e-commerce analytics, a critical research gap remains in accurately distinguishing between impulsive and planned (need-based) purchase behavior using multidimensional, high-velocity consumer data. Existing approaches often rely on either traditional econometric models that assume linear relationships or standalone machine learning classifiers with conventional hyperparameter tuning strategies such as grid search or random search. These methods face several limitations: (i) suboptimal parameter exploration in high-dimensional search spaces, (ii) vulnerability to local optima, (iii) limited generalization across heterogeneous consumer segments, and (iv) insufficient integration of behavioral theory into predictive pipelines.

As a result, current models frequently prioritize predictive accuracy without ensuring robustness, interpretability, and theoretical grounding, particularly when distinguishing psychologically distinct purchasing behaviors such as impulsive and planned decisions. Addressing these methodological and conceptual limitations requires an integrated framework that combines advanced optimization strategies, powerful ensemble learning techniques, and behaviorally informed feature design.

### Related work

The body of research on consumer behavior in e-commerce highlights the importance of trust mechanisms, big data analytics, and personalized engagement in forecasting and influencing purchase decisions. Lu and Liang have effectively developed a big data-based consumer behavior analysis model for international e-commerce, proving that extensive transactional and demographic datasets can reliably predict consumer preferences and buying patterns, especially a preference for electronics, clothing, and home goods^14^. Additionally, loyalty programs and personalization have become essential strategies for maintaining engagement in a quickly changing digital marketplace. Product quality, website usability, and strong after-sales support were found to be important drivers of customer satisfaction and competitive advantage in the online electronics sector^15^. Kant and Singh’s investigation of online shopping for electronic products also revealed that trust and convenience are crucial in consumer decision-making. The positive correlation between online purchasing decisions and consumer behavior is further supported by empirical evidence from Fitralisma and Ikhwan’s quantitative analysis in Brebes Regency^16^. They confirmed that knowledge of these behavioral determinants is crucial for e-commerce optimization by quantifying how consumer attitudes and behaviors impact purchase intent using regression techniques and validity, reliability, and normality tests. Similar to this, Riwayat, Susilawati, and Naqiah emphasized the importance of big data-driven strategies and sophisticated data analysis techniques for identifying market trends and buying patterns. They called for cooperation between academics, business professionals, and governmental organizations to improve data analytics capabilities, guarantee data security, and handle new opportunities and challenges in e-commerce^17^.

Explanations of consumer intent have also benefited greatly from theoretical frameworks. To forecast online shopping intentions, Lv’s intelligent e-commerce framework combines big data analytics, the technology acceptance model (TAM), and rational action theory^18^. Key performance indicators, including the order delivery ratio (95.2%), consumer behavior analysis ratio (92.6%), product quality ratio (97.6%), customer satisfaction ratio (95.9%), and demand prediction ratio (96.3%), all showed notable improvements after empirical validation. These outcomes demonstrate the effectiveness of combining cognitive-behavioral theories with sophisticated data analytics to create reliable predictive models, thereby enhancing e-commerce user experience and operational performance. Hwang’s subsequent work, which found that individualized interactions, excellent content, effective customer service, and transparent policies are crucial for boosting customer loyalty and engagement in the e-commerce landscape, has further emphasized the significance of content quality and omni-channel strategies^1^. An omni-channel strategy, which guarantees uniformity across digital and physical platforms, was found to be essential for meeting the preferences of digital consumers and efficiently utilizing technology. When taken as a whole, these studies show how complex consumer behavior is in online sales. They show that to effectively forecast and impact purchase decisions, a combination of big data approaches, trust-building techniques, personalization tactics, and theoretical frameworks is required. Researchers and practitioners can create more successful segmentation, targeting, and retention strategies by combining big data-driven predictive models with trust and convenience considerations, and by focusing on content quality and omni-channel consistency.

While prior studies demonstrate the value of big data analytics, trust mechanisms, and theoretical integration in understanding online purchasing behavior, three major limitations persist. First, most studies focus on general purchase intention rather than differentiating between impulsive and planned decision-making processes. Second, hyperparameter tuning is typically conducted using conventional or heuristic approaches without systematic comparison of advanced metaheuristic optimizers. Third, comparative evaluations across leading gradient boosting frameworks under unified optimization strategies remain limited. Consequently, there is insufficient empirical evidence regarding which hybrid configurations provide the most reliable and generalizable performance in real-world e-commerce settings.

### Objective blueprint

To address the methodological and conceptual gaps identified above, this study proposes an intelligent hybrid framework for predicting and explaining consumer purchase behavior in e-commerce environments. Specifically, the framework integrates two bio-inspired metaheuristic algorithms—Walrus Optimization Algorithm (WaOA) and Weevil Damage Optimization Algorithm (WDOA)—with three state-of-the-art gradient boosting classifiers: XGBoost, LightGBM, and CatBoost. The primary objective is not only to enhance predictive accuracy, but also to systematically evaluate whether advanced metaheuristic optimization improves convergence stability, robustness, and generalization when classifying impulsive and planned (need-based) purchase behavior.

In this study, two bio-inspired metaheuristic algorithms, WaOA and WDOA, are integrated to develop a novel optimization procedure that improves the performance of three top gradient boosting classifiers: CatBoost, XGBoost, and LightGBM. In contrast to more conventional hyperparameter search techniques like grid or random search, WaOA and WDOA more closely resemble natural foraging behaviors, better balancing exploration and exploitation and quickly converging to optimal parameter sets while avoiding local minima. This study builds hybrid models that dynamically modify learning rates, tree depths, and regularization parameters in response to incoming consumer behavior data by integrating these optimizers into each classifier’s training pipeline. This ensures reliable, highly accurate predictions even as data streams change. Although machine learning has been widely applied in consumer behavior analysis, several gaps remain. First, limited research has examined the systematic use of advanced bio-inspired optimization algorithms for hyperparameter tuning of gradient boosting models in retail datasets. Second, head-to-head comparisons of CatBoost, XGBoost, and LightGBM under identical metaheuristic optimization conditions are scarce, limiting practical guidance for model selection. Third, many existing studies rely on static or batch-processed datasets, overlooking the dynamic and high-dimensional nature of contemporary e-commerce data streams. Fourth, behavioral economic constructs are rarely operationalized as explicit predictive features, reducing interpretability and theoretical consistency. Finally, performance validation is often restricted to narrow experimental settings, raising concerns about robustness and generalizability. This study addresses these limitations through a unified hybrid framework, behaviorally informed feature engineering, and comprehensive comparative evaluation.

To guide the investigation, the study is structured around the following research questions:

#### RQ1

Can advanced gradient boosting models (XGBoost, LightGBM, and CatBoost) accurately distinguish between impulsive and planned purchase behavior using multidimensional e-commerce data?

#### RQ2

Does the integration of bio-inspired metaheuristic algorithms (Walrus Optimization Algorithm and Weevil Damage Optimization Algorithm) significantly improve hyperparameter tuning performance compared to conventional optimization approaches?

#### RQ3

Among the examined model–optimizer combinations, which configuration provides the most stable and generalizable classification performance?

#### RQ4

How do behavioral and loyalty-related features contribute to interpretability and predictive strength in distinguishing consumer decision types?

## Dataset

### Dataset overview

The “E-commerce Consumer Behavior Analysis Data” dataset used in this study was acquired from Kaggle. It has been carefully chosen to capture the various facets of customer behavior in online retail settings. The dataset includes a wide range of variables, including customer demographics, transactional histories, product preferences, engagement metrics, and decision-making indicators, to represent actual e-commerce interactions. This dataset provides a crucial empirical basis for analyzing e-commerce consumer behavior, enabling the identification of behavioral patterns, modeling purchase intent, and assessing factors that influence customer satisfaction and loyalty. Its design enables comprehensive investigation of insights at the individual and segment levels for a variety of product categories and online retail platforms. Tasks involving customer segmentation, marketing optimization, and predictive modeling are especially well-suited for this dataset. Data scientists, marketing analysts, and researchers can produce actionable intelligence to improve consumer targeting, product recommendation systems, personalized advertising, and churn-prevention tactics within e-commerce ecosystems by combining behavioral, psychological, and contextual characteristics.

The dataset contains 1000 samples categorized into four consumer decision-making classes. The class distribution is relatively balanced and is reported as follows:

- Impulsive: 248 samples (24.8%).
- Planned: 256 samples (25.6%).
- Need-based: 247 samples (24.7%).
- Wants-based: 249 samples (24.9%).

### Grouped feature categories

#### Demographic and socioeconomic information

These characteristics, which describe distinct customers, aid in segmentation and personalization:

- *Customer_ID*: An individual’s unique identification number.
- *Age*: The customer’s age is represented numerically.
- *Gender*: Gender identity (other, non-binary, female, or male).
- *Income_Level*: Low, Middle, or High financial standing.
- *Marital_Status*: Status of marriage (single, married, divorced, widowed).
- *Education_Level*: Academic experience (high school, bachelor’s, master’s, and doctoral degrees).
- *Occupation*: A job or vocation.
- *Location*: A city, region, or nation’s geographic location.

#### Purchasing behavior

This group offers comprehensive insights into transaction patterns and spending habits:

- *Purchase_Amount*: Each transaction’s monetary value.
- *Frequency_of_Purchase*: The quantity of monthly transactions.
- *Purchase_Category*: The kind of goods bought, such as apparel or electronics.
- *Purchase_Channel*: Buying method (online, in-store, mixed).
- *Time_of_Purchase*: The day and time of the transaction.
- *Payment_Method*: Payment method (Credit Card, Debit Card, PayPal, Cash, Other).
- *Payment_Frequency*: Structure of payments (one-time, subscription, installments).

#### Customer engagement and loyalty

These characteristics evaluate the long-term connection with platforms and brands:

- *Brand Loyalty*: Brand attachment level (1–5).
- *Customer_Loyalty_Program_Member*: Membership in the loyalty program (True/False).
- *Engagement_with_Ads*: High, Medium, Low, and None interactions with marketing content.

#### Product feedback and post-purchase experience

Utilized to assess customer satisfaction and product performance:

- *Product_Rating*: A customer’s evaluation of the things they have bought on a scale of 1 to 5.
- *Customer_Satisfaction*: Total satisfaction rating on a scale of 1 to 10.
- *Return_Rate*: The proportion of items that are returned.
- *Discount_Used*: Indicates whether a discount was applied (True/False).

#### Decision-making process

Represents the behavioral and cognitive components of purchase intent:

- *Time_to_Decision*: The number of days from product consideration to purchase.
- *Time_Spent_on_Product_Research*: The amount of time spent assessing a product.
- *Purchase_Intent*: The buying motivation (Impulsive, Planned, Need-based, Wants-based).

#### External and contextual influences

These elements demonstrate the impact of contextual elements and outside stimuli:

- *Social Media Influence*: Social media platforms’ degree of influence (High, Medium, Low, or None).
- *Discount_Sensitivity*: The degree to which a product is sensitive to discounts (very sensitive, somewhat sensitive, not sensitive).
- *Shipping_Preference*: Standard, Express, or No Preference are the preferred shipping options.
- *Device_Used_for_Shopping*: The gadget (tablet, smartphone, or desktop) used to make the purchase.

**Fig. 1:**
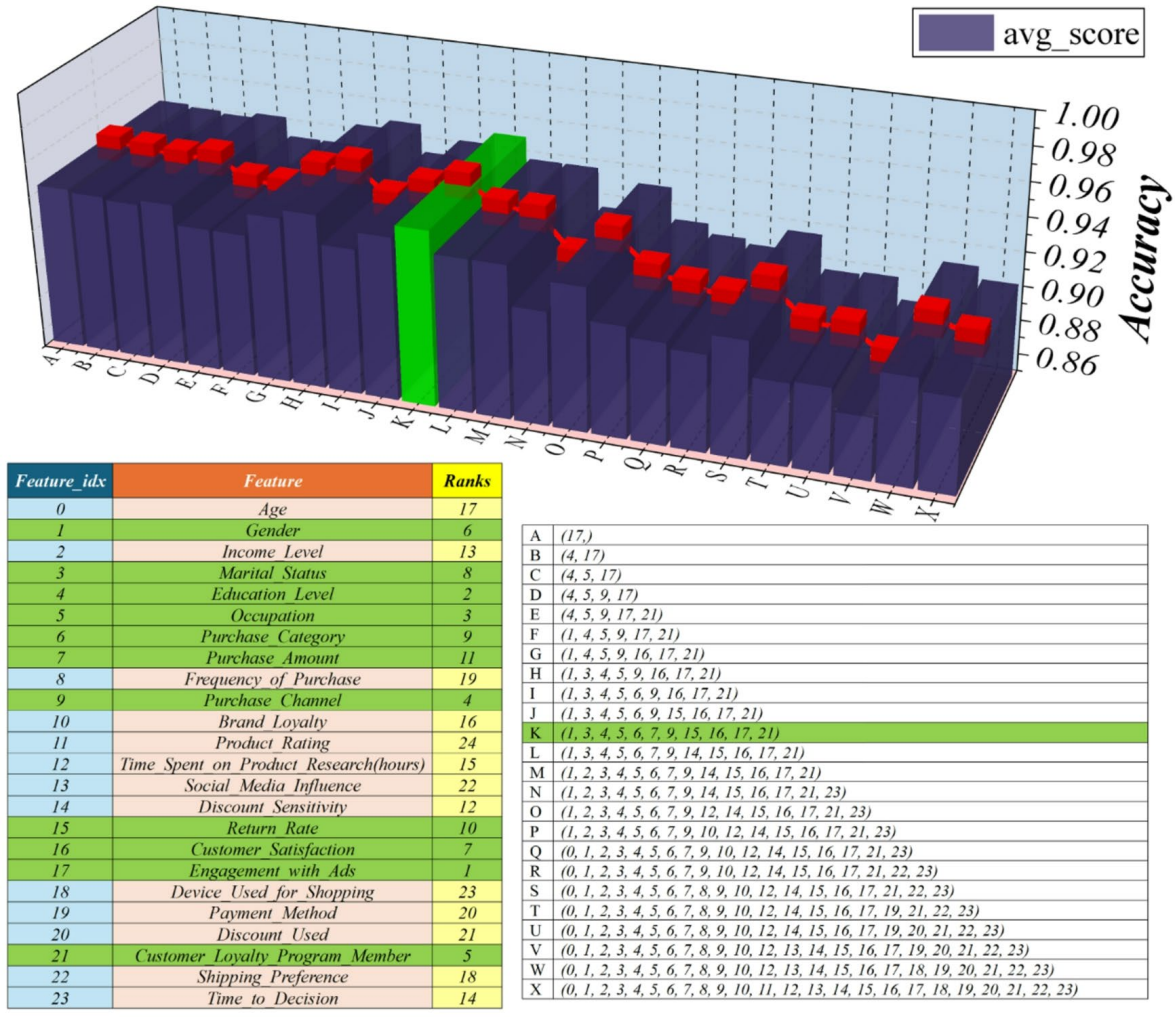
3D bar chart displaying the importance scores of chosen features according to their impact on the model’s performance.

The feature importance scores based on the e-commerce consumer behavior are displayed in a 3D bar chart in Fig. 1, with an emphasis on how well they predict purchase intent. With the highest importance score, Product_Rating is the most significant variable. This suggests that when determining whether to make a purchase, customers give product ratings considerable weight. This feature’s prominence aligns with well-established e-commerce behavior, where consumers often rely on others’ experiences to reduce uncertainty and inform their decisions. Its high predictive value underscores the importance of perceived product quality in shaping customer choices. This insight underscores the importance of online businesses upholding high product standards and promoting genuine customer reviews. The model’s capacity to recognize Product_Rating as a critical component attests to its efficacy in identifying significant patterns in consumer behavior and supports focused tactics for improving user experience and raising conversion rates.

**Fig. 2:**
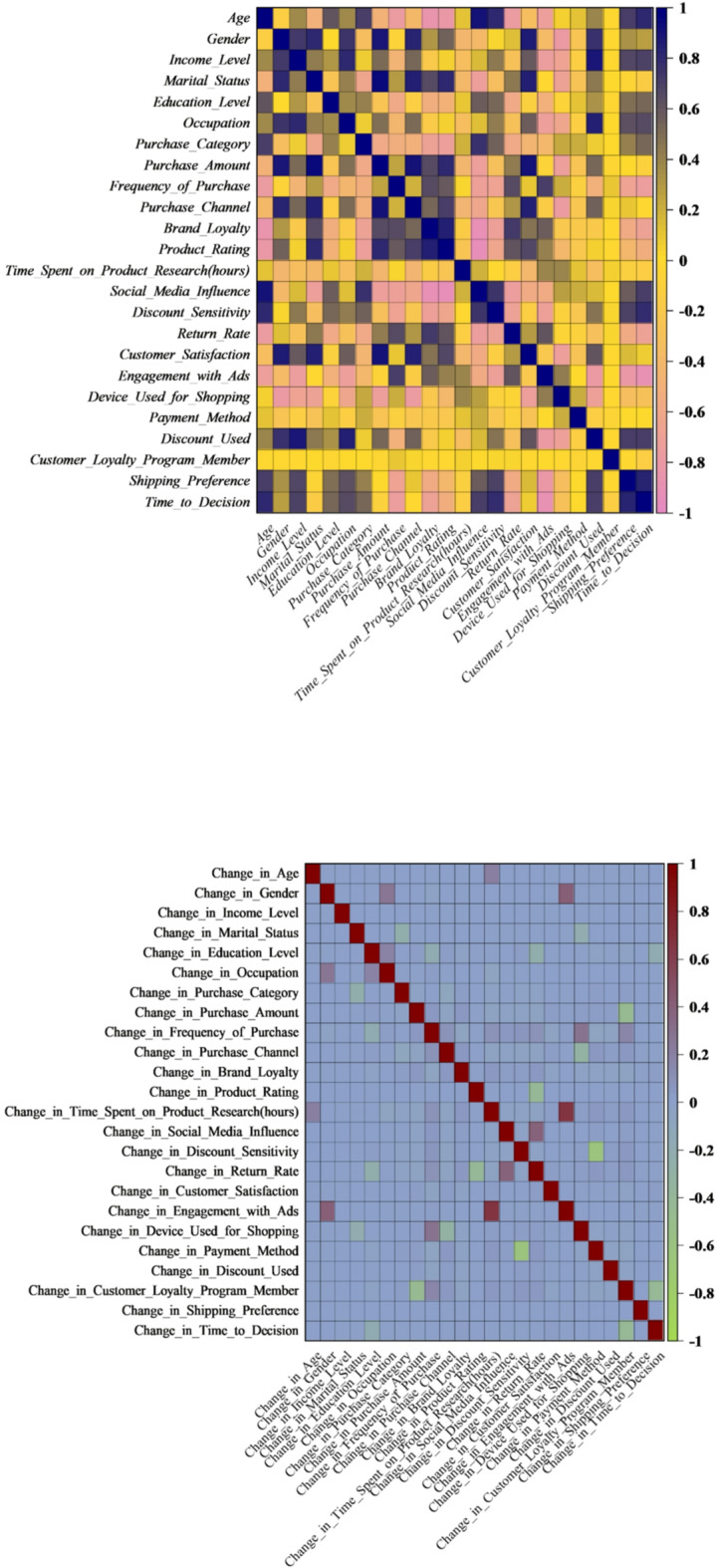
The correlation plot presents counterfactual explanations, highlighting how minimal changes to input features can alter the model’s predictions while preserving logical associations within the data.

The key outcome variable in the e-commerce consumer behavior dataset, according to Fig. 2, is Purchase Intent. It shows strong positive correlations with Customer_Satisfaction, Product_Rating, Discount_Used, Customer_Loyalty_Program_Member, Frequency_of_Purchase, and Brand_Loyalty. These connections highlight how crucial customer satisfaction, product assessment, and loyalty are in influencing purchase decisions. On the other hand, Time_to_Decision shows a strong inverse relationship with Purchase_Intent, indicating that shorter decision time is associated with a higher likelihood of purchase. The interdependencies among feature changes required to alter purchase outcomes plausibly are revealed by further analysis using the “Change_in” correlation matrix, which is intended to support counterfactual explanations. In particular, for a shift from non-purchase to purchase behavior to occur, decreases in Time_to_Decision must be accompanied by increases in Customer_Satisfaction and Product_Rating. This pattern highlights the necessity of consistent simultaneous adjustments when modeling behavioral shifts and reflects realistic consumer dynamics. These findings directly affect e-commerce strategy optimization. Businesses can more effectively create interventions like targeted promotions, enhanced customer service, and loyalty incentives by identifying the critical factors whose changes have the greatest impact on Purchase Intent. By ensuring that suggested changes maintain their contextual validity, counterfactual reasoning improves the accuracy of behavioral predictions and facilitates data-driven decision-making in customer interaction.

#### Data quality and class distribution

Prior to model development, the dataset was examined for missing values and class imbalance. The analysis confirmed that no missing or null entries were present across the feature space, eliminating the need for imputation or data cleaning procedures.

In terms of class distribution, the dataset exhibited a relatively balanced structure between impulsive and planned (need-based) purchase categories. The proportions of samples across classes were sufficiently similar to avoid a severe imbalance that could bias model training. Therefore, no resampling techniques (e.g., SMOTE, undersampling, or class-weight adjustments) were required.

Given this distribution, standard evaluation metrics such as accuracy, precision, recall, and F1-score were considered appropriate for performance assessment. The use of class-specific F1-scores further ensures that model performance is evaluated consistently across both decision categories, mitigating the risk of misleading accuracy results when minor class disparities are present.

### Data preprocessing and feature engineering

Prior to model training, several preprocessing steps were performed to ensure data quality, consistency, and compatibility with the machine learning algorithms.

#### Data cleaning

An initial inspection confirmed that the dataset contained no missing or null values across features. Therefore, no imputation techniques were required. Duplicate records were examined, and none were identified. The Customer_ID variable was removed prior to modeling, as it serves solely as a unique identifier and does not contribute predictive value.

#### Target variable preparation

The target variable, Purchase_Intent, originally included four categories: Impulsive, Planned, Need-based, and Wants-based. For this study, the classification task focused on distinguishing impulsive from planned (need-based) purchase behavior. Accordingly, related categories were consolidated where appropriate to create a binary classification framework, ensuring conceptual clarity and model interpretability.

#### Encoding of categorical variables

Categorical features were transformed into numerical representations suitable for gradient boosting models:

- Binary variables (e.g., Loyalty Program Membership, Discount Used) were encoded using binary encoding (0/1).
- Nominal categorical variables (e.g., Gender, Occupation, Location, Payment Method, Device Used) were encoded using one-hot encoding to prevent ordinal bias.
- Ordinal variables (e.g., Income Level, Education Level, Engagement with Ads, Discount Sensitivity) were encoded using ordinal mapping that preserved their inherent ranking structure.

For CatBoost, which natively handles categorical features, the model was provided with categorical indices to leverage its internal encoding mechanism.

#### Encoding of categorical variables

Since gradient boosting models are tree-based algorithms and are generally insensitive to feature scaling, no normalization or standardization was applied. Continuous numerical variables, such as Age, Purchase Amount, Frequency of Purchase, Time to Decision, and Time Spent on Product Research, were used in their original scales.

#### Train–Test split

The dataset was partitioned into training and test sets using stratified sampling to preserve class distribution across both sets. Specifically, 70% of the data were allocated for training and 30% for testing. Stratification ensured balanced representation of impulsive and planned classes in both subsets.

## Classification models and hyperparameter optimization framework

### Categorical boosting (CatBoost)

**Fig. 3:**
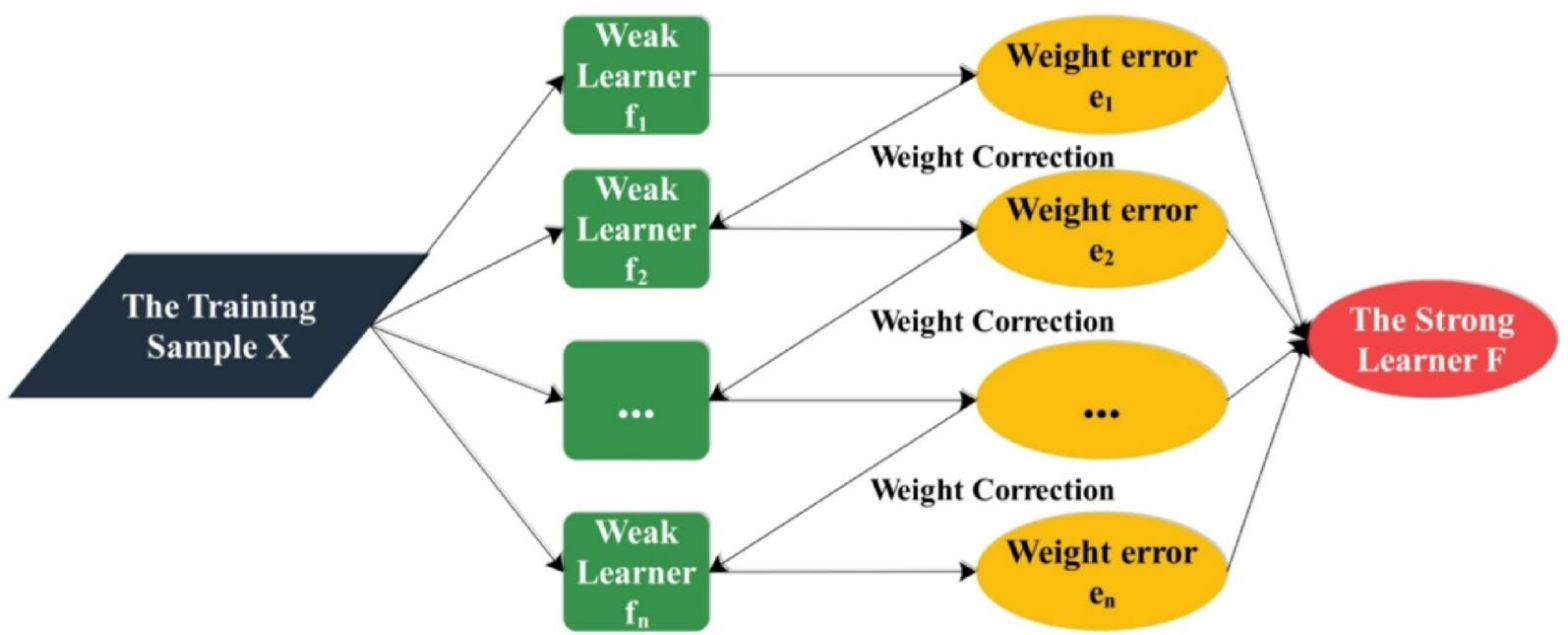
Structure of CatBoost.

CatBoost is an open-source ML algorithm developed by Yandex and widely used in Python and R. It is based on the Gradient Boosting Decision Tree (GBDT) framework and uses symmetric decision trees as its learning structure. Among CatBoost’s defining features is its exceptional capacity to work with categorical features directly and efficiently, and its high applicability in e-commerce consumer behavior analysis stems largely from the abundance of categorical variables in most e-commerce datasets^19^. These variables include user attributes, product categories, browser devices, and transaction channels. CatBoost is specifically designed to extract significant patterns from data of this nature without needing extensive preprocessing and manual encoding. While accuracy combined with resistance to overfitting is crucial for making dependable insights in consumer behavior analysis, CatBoost stabilizes learning through minimizing gradient bias and prediction shift common to conventional boosting algorithms and takes a novel approach to working with categorical data as opposed to using simple statistical measures for node-splitting in decision trees (which introduces bias when data distribution differs during training and testing)^20^. CatBoost utilises prior distributions to stabilize learning, particularly in dynamic e-commerce contexts where user behavior patterns change rapidly due to marketing initiatives, seasonality, or external factors. CatBoost’s ability to automatically generate novel feature combinations from existing categorical inputs makes it well-suited to uncovering latent associations between consumer attributes and buying behavior. These feature combinations enable more complex decision-making processes, such as how a customer’s device interacts with their location and product preference, and queues up intentions to buy, to be better captured by the model. With its high accuracy and immunity to overfitting, combined with its native capacity for handling categorical data, CatBoost is a viable and pragmatic tool for e-commerce consumer behavior analysis, enabling more accurate targeting, personalization, and strategic decision-making^21^. Figure 3 presents the CatBoost model flowchart below.

### Extreme gradient boosting (XGBoost)

**Fig. 4:**
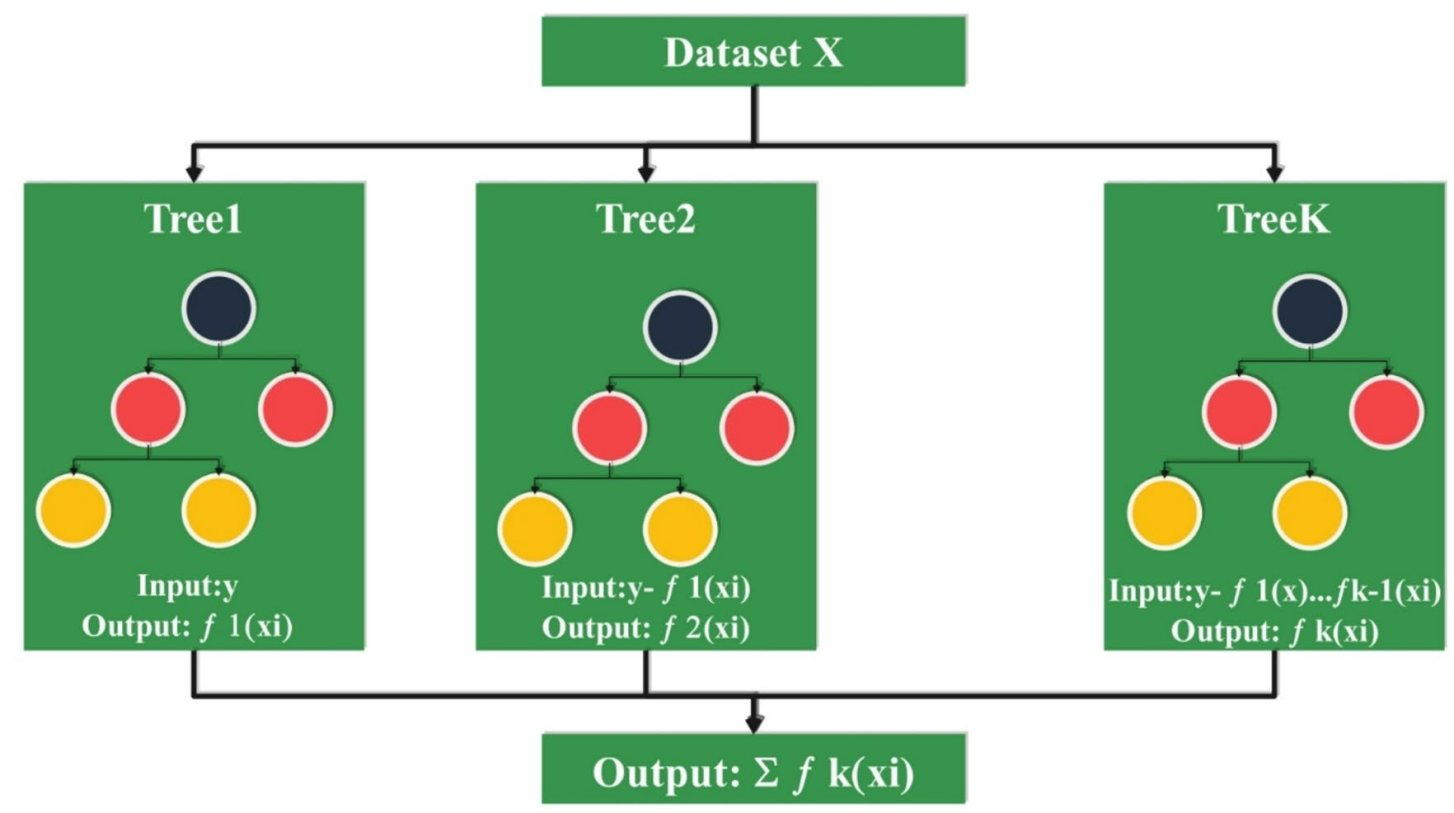
Structure of XGBoost.

XGBoost is a highly versatile and popular ML algorithm with strong performance in supervised learning applications such as regression and classification. What makes XGBoost very desirable among data scientists is its high computational efficiency and its ability to handle large, complex datasets. This makes it appropriate for applications such as tracking consumer behavior within e-commerce contexts. In e-commerce consumer behavior analysis, datasets commonly include a vast array of features, including purchase history, browsing habits, demographic information, product category data, and timestamps. XGBoost operates by constructing an ensemble of decision trees where each tree is trained to correct the mistakes made by its predecessors in a sequential boosting algorithm. This allows the model to learn more complex patterns and feature interrelations, which are critically necessary for understanding and predicting customer actions, interests, and purchase intention^22,23^. One advantage of XGBoost is that it is capable of reducing prediction error and controlling model complexity as well. This is especially desirable when analyzing consumers since overfitting easily takes hold due to high dimensionality and volatility in datasets. The algorithm incorporates regularization methods to prevent overfitting, ensuring the model generalizes well to new data and yields more reliable predictions in real-world applications, such as targeted marketing, product suggestion, or customer group segmentation. XGBoost is also very effective at handling missing data and can produce feature importance rankings, enabling analysts to identify the variables that most affect consumer decisions. This is particularly convenient for firms interested in maximizing user experience and personalizing promotions based on data-driven insights. Generally speaking, XGBoost’s reliability, speed, and accuracy make it an important modeling and prediction tool for consumer behavior on e-commerce platforms^24^. Figure 4 provides the structure of the XGBoost model.

### Light gradient boosting machine (LightGBM)

**Fig. 5:**
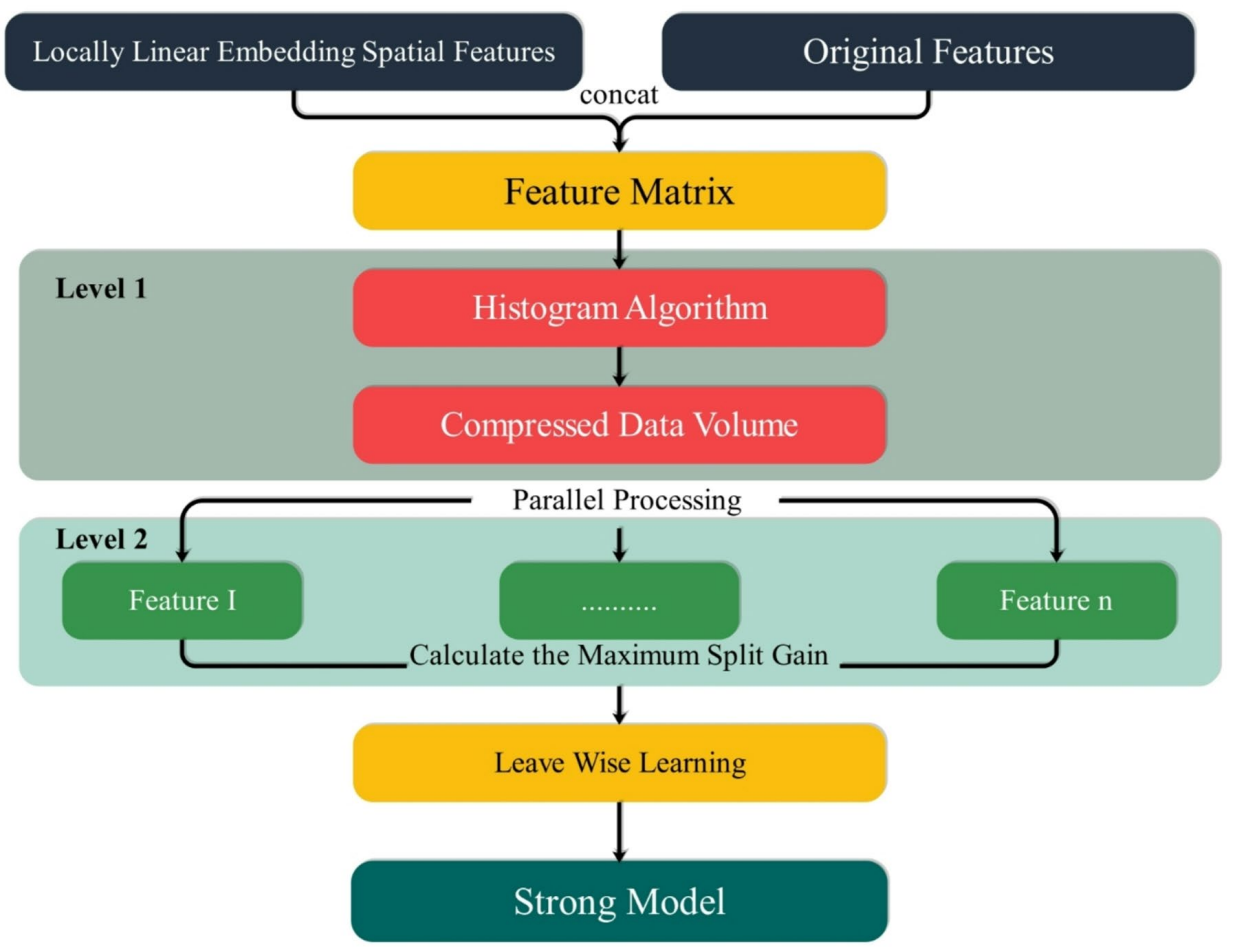
Structure of the LGB.

LightGBM is an extremely efficient and scalable decision tree-based ensemble learning algorithm well-suited to handling large datasets like those in the E-commerce Consumer Behavior Analysis Data. For e-commerce, where understanding consumer preferences, buying behavior, and product interactions is essential, LightGBM enables rapid, accurate processing of high-dimensional data, providing quick, actionable insights for customer segmentation, recommendation systems, and personalization. One major advantage of LightGBM is gradient-based one-sided sampling (GOSS), which intelligently repeats data examples with greater error (gradients) while randomly sampling from less informative data points^25^. This is particularly beneficial in consumer behavior data, as some user events, for instance, an unexpected interest in a novel product category or abandonment in a shopping cart, hold greater forecast values than common clicks or view events. By focusing on such behavior, LightGBM improves model learning efficiency without sacrificing accuracy. Another innovative feature is Exclusive Feature Bundling (EFB), which bundles infrequently active features together as one. Many e-commerce datasets have sparse features, for instance, one-hot encoded product categories, demographics, and browse actions. With reduced active features and faster model fitting, EFB enables analysts to easily explore customer journeys and uncover hidden consumer behavior patterns. LightGBM also uses a histogram-based strategy to discretize continuous consumer data, such as time on site, session duration, or price sensitivity, into discrete bins. This method reduces memory consumption and accelerates optimal decision splits, enabling real-time computation on user engagement and purchase triggers. Its parallel and distributed computing support further makes LightGBM an ideal solution for e-commerce websites with millions of user activities. Rather than horizontally partitioning a dataset across machines and creating a communication-intensive, complex process, LightGBM fits entire datasets locally and sends only the necessary results to avoid latency. This enables online merchants and platforms to train behavior prediction models more quickly and expand them to vast consumer databases with greater ease. Moreover, LightGBM utilizes scatter-gather reduction and vote-based data parallelism that lessens server-to-server communication overhead. This enables smooth processing even when predicting high-volume events such as flash sales, holiday trends, or campaign performance^26,27^. These mechanisms keep the model responsive and nimble, even as consumer data volume and complexity increase. In general, LightGBM’s design is ideal for e-commerce consumer behavior analysis and provides a strong framework for extracting meaningful insights from user data, predicting purchase intent, and personalizing the online shopping experience in real time. Figure 5 presents the LightGBM model’s flowchart.

### Walrus optimization algorithm (WaOA)

**Fig. 6:**
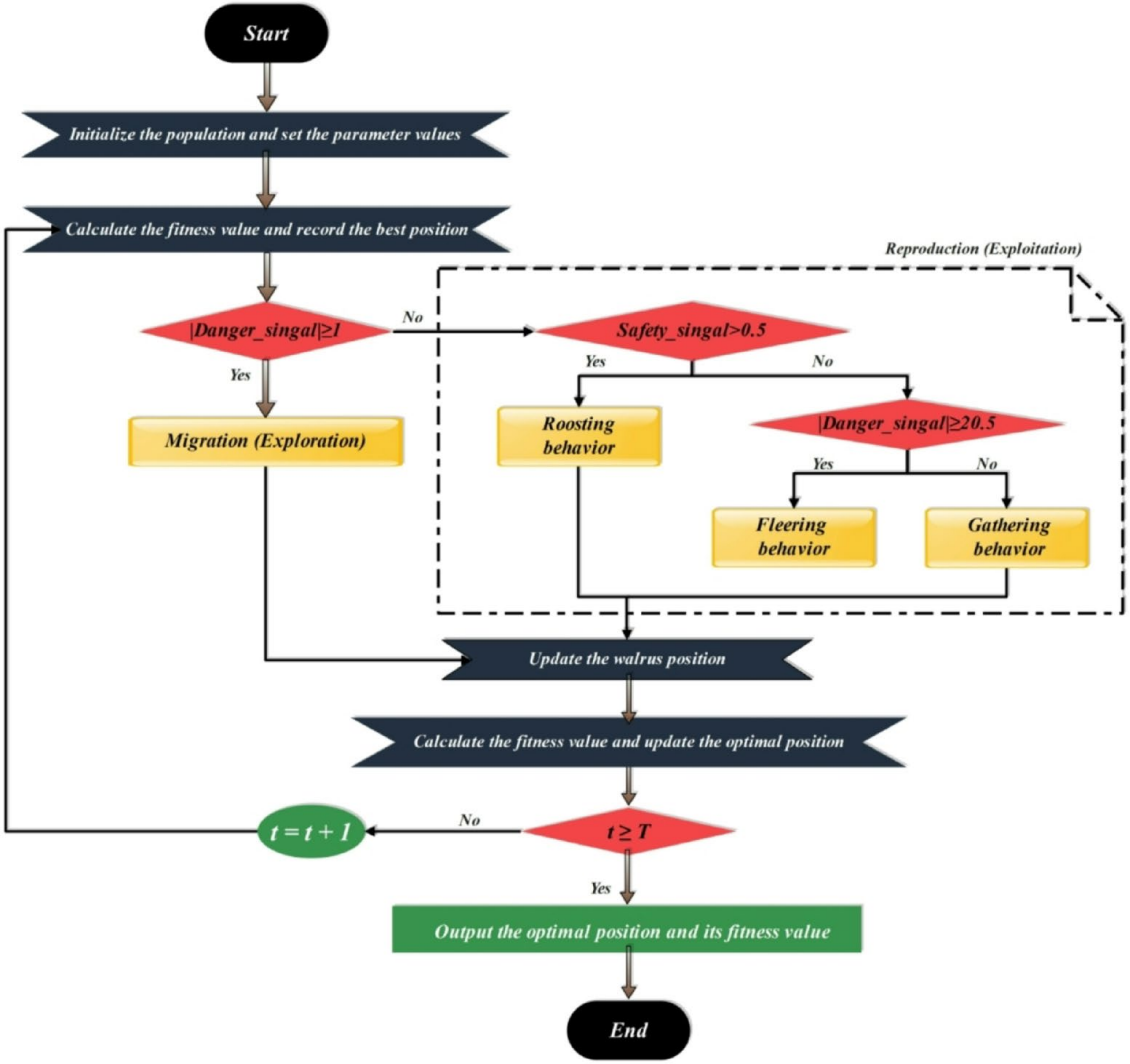
Flowchart of WaOA.

The WaOA is a novel metaheuristic approach based on walruses’ natural behavior, their sensitivity, and their group awareness of their surroundings. In nature, walruses use their senses and interplay among adults, juveniles, and females to effectively respond to opportunities and threats. The WaOA imitates their behavior to guide a population of potential solutions through an investigation space, balancing exploration and exploitation throughout the optimization process^28^. Used in the application of E-commerce Consumer Behavior Analysis Data, it aids in easily navigating e-commerce data through its intricate patterns and consumer relationships. Its implementation starts with producing a diverse generation of initial candidate solutions representing various possible patterns or model parameters. This diversity is crucial for achieving comprehensive coverage of the behavioral space in e-commerce data, which encompasses a wide range of features, from demographics to browsing activity and buying trends. At its core are “danger” and “safety” signals that mimic how walruses respond to their surroundings and interact with each other and their environment^29^. In consumer behavior data analysis, they act as indicators stimulating movement and direction in the search space towards areas less likely to hold optimal consumer behavior patterns and model configurations (danger signal) and towards areas more likely to explain and predict consumer actions (safety signal). Over time, as it refines its solution space, it overrules the influence of the danger signal and increases the impact of its safety signal, allowing for a more selective final refining process into consumer behavior insights. This adaptive balancing enables WO to escape local optima common in complex e-commerce data, thereby improving the accuracy and appropriateness of behavioral models continuously. By incorporating a nature-based strategy that draws on walruses’ responses to environmental stimuli, the algorithm design optimizes and fully exploits e-commerce data, including its intricate patterns and consumer relationships. This enables the easy identification of specific buying patterns, trends, and consumer preferences with greater accuracy^30^. In Fig. 6, the WaOA flowchart is presented.

### Weevil damage optimization algorithm (WDOA)

WDOA is a bio-inspired metaheuristic that simulates the destructive activities of weevils in nature and adapts those concepts to tackle intricate optimization challenges. Initiated based on how weevils affect agricultural commodities, WDOA operates through a swarm-based strategy in which every weevil is a possible solution to a problem. When applied to E-commerce Consumer Behavior Analysis, WDOA is well-suited to optimize various tasks, ranging from feature selection to segmentation approaches to fine-tuning ML algorithms that predict consumers’ actions. Every single weevil in the algorithm is a distinct set of consumer behavior parameters, e.g., duration on site, frequency of purchase, preferred product category, discount sensitivity. The goal is to identify patterns or sets of variables with strong influence, yielding valuable insights or highly accurate predictive models. The algorithm evaluates them based on their ability to achieve a target goal, such as maximizing accuracy in predicting repeat purchases or enhancing high-value customer classification. WDOA applies three behavioral mechanisms: flight power, snout power, and damage power.

In an e-commerce context, flight power measures how well an algorithm can broadly navigate diverse customer profiles and behavioral attributes to identify novel trends. Snout power represents how the algorithm can differentiate and hone in on consumer intelligence data to fine-tune prediction models. Damage power drives the algorithm to pursue optimal solutions forcefully by focusing on behavioral attributes with the highest propensity to influence ecommerce results, such as conversion rates and shopping cart abandonment. The algorithm runs through a sequence of iterations in which each weevil (solution) is tested and evaluated according to its performance in understanding and forecasting consumer behavior. At each step of the iterative process, the top-performing configurations are retained and forwarded to subsequent steps, ensuring continuous improvement and convergence toward an optimal solution. WDOA also adds flexibility via mechanisms such as mutation and environmental responsiveness to increase diversity among the solution population and prevent being trapped in poor areas in the solution space. In e-commerce contexts, this enables the algorithm to remain versatile and effective, regardless of changing datasets, user segments, and market trends. By emulating weevils’ adaptive survival strategies, WDOA emerges as a highly effective analytical tool for e-commerce consumer behavior. Businesses use it to delve deeper into customer data to enhance decision-making tactics and gain a competitive advantage by identifying patterns that are not easily traceable using conventional methods^30^.

### Performance evaluators

As Table 1 explains, four primary evaluation metrics — Accuracy, Precision, Recall, and F1-Score — are used in the e-commerce consumer behavior analysis to thoroughly evaluate how well the created classification models predict various consumer purchase patterns (impulsive, need-based, planned, and want-based). The percentage of correctly classified instances (both true positives and true negatives) in relation to the total number of predictions the model produces is known as accuracy. In e-commerce, where classes like high-intent buyers may be underrepresented, accuracy can have limited interpretive value even though it offers a broad measure of correctness. A high overall accuracy could mask inadequate detection of these important but less common behaviors. Precision is computed as the ratio of true positive predictions to the total of true positive and false positive predictions in order to overcome this constraint. In this field, accuracy is crucial for ensuring that marketing initiatives, such as targeted ads for potential customers, are distributed effectively, thereby reducing spending on non-converting users.

On the other hand, recall (also known as sensitivity) measures the model’s ability to identify every real buyer by dividing the number of true positive predictions by the total of true positives and false negatives. To capture as many real buyers as possible and lessen the chance of missing out on important customer segments, high recall is crucial. The F1-score is used as the harmonic mean of precision and recall because of the intrinsic trade-off between the two, where gains in one frequently result in decreases in the other. By penalizing models that display disproportionate rates of false positives or false negatives, this composite metric offers a fair evaluation. In real-world applications, a strong F1-Score shows that the model successfully separates complex consumer behaviors without unduly prioritizing either recall or precision. Taken together, these metrics provide a comprehensive framework for assessing classification performance in an online retail environment. The study guarantees that predictive models not only achieve high overall accuracy but also strike a careful balance between correctly identifying actual buyers and avoiding false-positive classifications by incorporating these evaluators. Strategies like dynamic pricing, personalized recommendation engines, and customer retention programs all benefit significantly from this fair assessment.

**Table 1:** Formulas for key classification performance evaluation metrics.

Metric	Formula	Description
*Accuracy*	$\:\frac{TP+TN}{TP+TN+FP+FN}$	Proportion of total correct predictions out of all predictions made.
*Precision*	$\:\frac{TP}{TP+FP}$	Proportion of positive predictions that were actually correct.
*Recall*	$\:\frac{TP}{TP+FN}$	Proportion of actual positives that were correctly predicted.
*F1-score*	$\:2\times\:\frac{\mathrm{P}\mathrm{r}\mathrm{e}\mathrm{c}\mathrm{i}\mathrm{s}\mathrm{i}\mathrm{o}\mathrm{n}\times\:\mathrm{R}\mathrm{e}\mathrm{c}\mathrm{a}\mathrm{l}\mathrm{l}}{\mathrm{P}\mathrm{r}\mathrm{e}\mathrm{c}\mathrm{i}\mathrm{s}\mathrm{i}\mathrm{o}\mathrm{n}+\mathrm{R}\mathrm{e}\mathrm{c}\mathrm{a}\mathrm{l}\mathrm{l}}$	Harmonic mean of Precision and Recall, balancing both in a single metric.

TP ,  True Positive; TN, True Negative;  FP, False Positive; FN, False Negative.

### Experimental setup

To ensure transparency and reproducibility, the hyperparameter configurations reported in Table 2 were carefully verified against the official Python implementations of XGBoost, LightGBM, and CatBoost. All parameter names match the respective library documentation, and the reported values reflect the optimal solutions returned by the metaheuristic search rather than predefined defaults.

The hyperparameter search spaces were defined as follows: maximum tree depth was explored over a broad range to allow the optimization algorithms sufficient flexibility to identify deep interaction structures; learning rate values were sampled over a continuous interval; max_bin and thread_count were tuned within the allowable bounds defined by the respective libraries. These ranges were intentionally wide to prevent premature restriction of the search space, which could otherwise bias optimization results.

All experiments were conducted in Python on a system equipped with an Intel Core i7 (11th Generation) processor and 64 GB of RAM, running Windows 11 (64-bit). Default multi-threading capabilities of each library were enabled. The runtime required for each hybrid configuration reflects the iterative nature of the metaheuristic algorithms, which evaluate multiple candidate solutions across generations. The total execution times were as follows:

- XGB+WaOA: 1620 s.
- XGB + WDO: 2085 s.
- XGB: 1.5 s.
- LGB + WOA: 1296 s.
- LGB + WDO: 1668 s.
- LGB: 1.2 s.
- CAT + WOA: 1836 s.
- CAT + WDO: 2363 s.
- CAT: 1.7 s.

The substantial difference between baseline and optimized runtimes is expected, as metaheuristic optimization involves repeated model training across multiple candidate hyperparameter sets. Stopping criteria were defined based on a fixed number of generations and population size, ensuring consistent computational budgets across all hybrid models.

## Result

The hyperparameter configurations of several hybrid models used to analyze e-commerce consumer behavior are shown in Table 2, highlighting the importance of parameter tuning for maximizing model performance. The learning rate is one of the most important hyperparameters, as it determines the model’s effectiveness and predictive power. To quickly adapt to intricate patterns in customer engagement metrics and purchase behavior, the XGWD model, for example, employs an exceptionally high learning rate of 0.999. On the other hand, models like LGWO and XGWO use lower learning rates, roughly 0.74–0.76. This shows a conscious balance between model stability and convergence speed, reducing the risk of overfitting given the inherent heterogeneity and noise in e-commerce datasets. This variance in the choice of learning rate highlights the importance of adjusting the model’s sensitivity to behavioral cues from online transactions. While an overly conservative learning rate may hinder the model’s ability to capture complex consumer decision-making processes, an overly high rate may lead the model to overfit fleeting trends or outliers. In addition to learning rate, tree complexity parameters, such as maximum depth in XGBoost-based models and the number of leaves in LightGBM variants, also affect how well the model captures complex relationships between important features, such as product categories, customer demographics, and loyalty program participation. To improve the predictive accuracy and generalizability of the model in e-commerce consumer behavior analysis, careful calibration of these hyperparameters is essential. More accurate predictions of purchase intent and more successful personalization tactics are enabled by optimized models’ superior ability to identify subtle yet significant patterns in customer data. Thus, in addition to improving model performance, the hyperparameter tuning process enhances the interpretability of consumer behavior insights that are crucial for data-driven marketing and CRM campaigns.

To ensure transparency and reproducibility, the search ranges used during hyperparameter optimization were predefined based on the allowable ranges of the respective machine learning libraries. The WaOA and WDOA explored the following parameter spaces during the tuning process.

For XGBoost, the search ranges included:

- n_estimators: 100–1000.
- max_depth: 10–1000.
- learning_rate: 0.001–1.0.
- colsample_bytree: 0.001–1.0.
- subsample: 0.1–1.0.
- reg_alpha: 0–1.
- reg_lambda: 0–1.

For LightGBM, the following ranges were explored:

- n_estimators: 50–1000.
- max_depth: 10–500.
- num_leaves: 10–700.
- learning_rate: 0.001–1.0.
- max_bin: 100–800,000.

For CatBoost, the optimization search space included:

- learning_rate: 0.001–1.0.
- bagging_temperature: 0–1.
- random_strength: 0–1.
- l2_leaf_reg: 1–1000.
- max_ctr_complexity: 1–10.
- fold_len_multiplier: 1–1000.
- thread_count: 1–1000.

The optimal hyperparameter values obtained after optimization are presented in Table 2.

**Table 2:** The hyperparameters of the models, along with their assigned values.

Hyperparameter	Hybrid Models					
	XGWD	XGWO	LGWD	LGWO	CAWD	CAWO
n_estimators	483	786	119	796	–	–
max_depth	999	357	148	174	–	–
learning_rate	0.999	0.755	0.361	0.747	0.258	0.354
colsample_bytree	0.001	0.677	–	–	–	–
subsample	0.999	0.883	–	–	–	–
reg_alpha	0.417	0.446	–	–	–	–
reg_lambda	0.158	0.271	–	–	–	–
num_leaves	–	–	213	629	–	–
max_bin	–	–	761,000	657,000	–	–
bagging_temperature	–	–	–	–	0.671	0.581
random_strength	–	–	–	–	0.724	0.999
l2_leaf_reg	–	–	–	–	415	645
max_ctr_complexity	–	–	–	–	2.97	8.33
fold_len_multiplier	–	–	–	–	369	685
thread_count	–	–	–	–	437	849

**Fig. 7:**
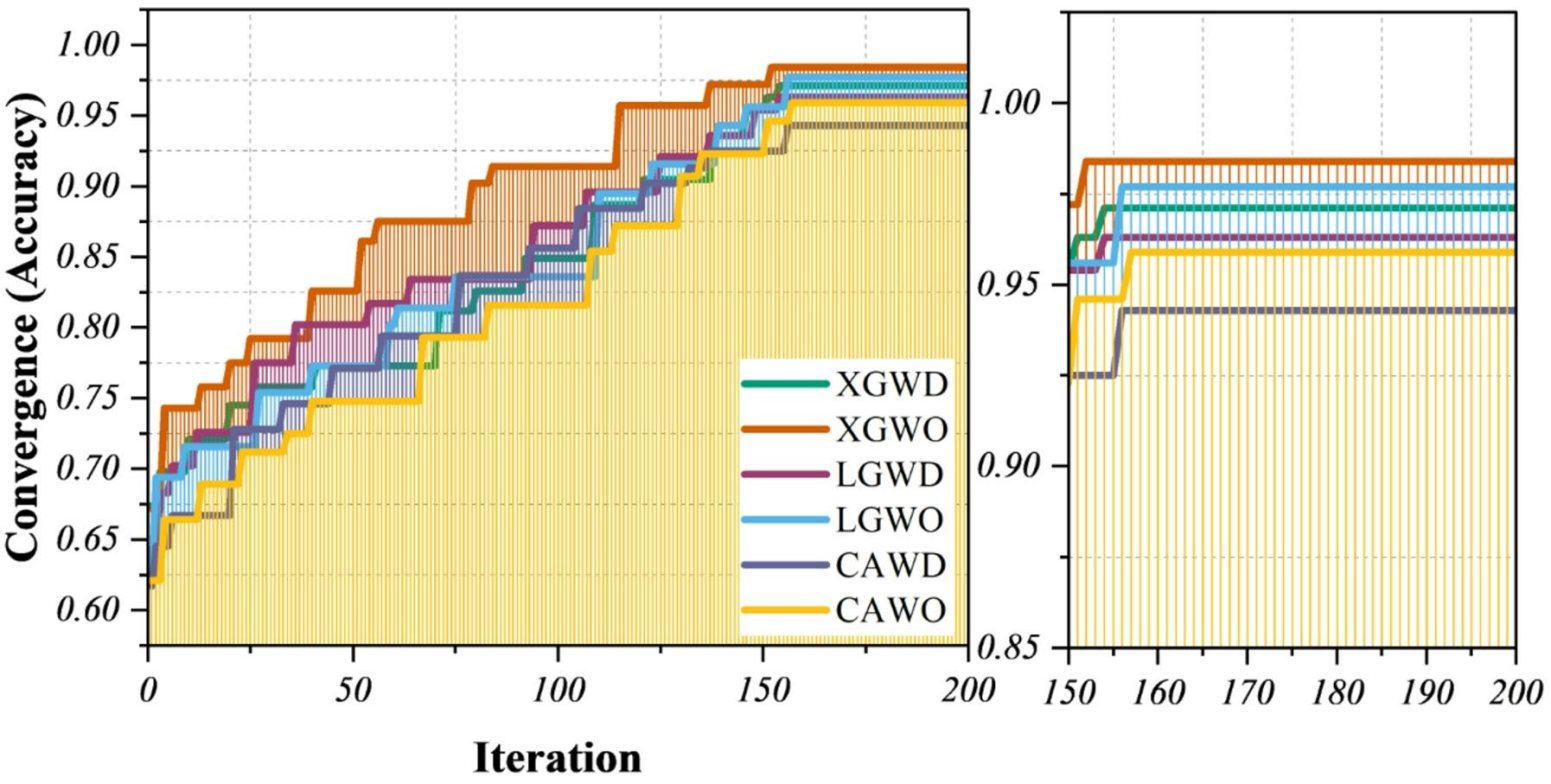
Line plot for the optimization process’s convergence over iterations, showing continuous improvement in model performance.

With special attention to the superior performance of the XGBoost model optimized by the XGWO, Fig. 7 shows the convergence process of several hybrid models optimized using sophisticated metaheuristic algorithms. The best method for simulating customer behavior in e-commerce settings is a hybrid model that achieves the highest accuracy and fastest convergence. Model accuracy, a crucial metric for evaluating the predictive power of models analyzing consumer behavior data, is plotted on the y-axis, while the x-axis shows the number of optimization iterations, reflecting the gradual tuning of model hyperparameters. In contrast to all other tested configurations, the XGWO hybrid model achieves near-optimal accuracy (approximately 0.98), demonstrating its accuracy and resilience in capturing the intricate buying patterns, preferences, and engagement signals typical of e-commerce consumer datasets. This improved accuracy and faster convergence suggest that the XGWO model effectively searches the hyperparameter space to find the best setup, thereby improving the model’s capacity to learn from high-dimensional, heterogeneous e-commerce data. This hybrid approach is especially well-suited to model the complexity of consumer behavior, which is characterized by non-linear interactions between features like browsing history, product categories, and transaction frequencies. The results will be more accurate predictions for applications such as customer segmentation, personalized recommendations, and targeted marketing.

Additionally, the clear performance difference between the XGWO model and other hybrid models (such as those based on LightGBM and CatBoost with different optimizers) underscores the importance of choosing both an appropriate base model and an efficient optimization algorithm suited to the specific patterns in e-commerce consumer behavior data. XGBoost’s generalizability and resistance to overfitting are improved by WaOA’s ability to adjust model parameters. This is important in real-world e-commerce scenarios where customer behavior is dynamic and ever-changing. In conclusion, the XGWO hybrid model’s superior accuracy and convergence behavior confirm its effectiveness as a predictive tool in the analysis of consumer behavior in e-commerce. The performance of this model demonstrates the vital role that advanced hybrid modeling and optimization techniques play in releasing actionable insights from intricate consumer data, which in turn helps businesses enhance customer satisfaction and inform strategic decision-making.

Accuracy, precision, recall, and F1-Score are key metrics used to assess the performance of ML models applied to e-commerce consumer behavior data during both the training and testing stages, as shown in Table 3. When it comes to forecasting consumer behavior, the hybrid XGBoost optimized with WaOA (XGWO) is the most successful model tested. With training and testing accuracy of 0.989 and 0.973, respectively, the XGWO model outperforms all other single and hybrid models. This improved performance demonstrates how well the hybrid approach captures the dynamic, complex nature of consumer behavior on e-commerce platforms. It does this by combining XGBoost’s powerful gradient boosting with the advanced Walrus Optimization Algorithm for hyperparameter tuning. XGWO refines the model to better detect patterns like purchase intent, browsing habits, and churn likelihood by effectively examining the hyperparameter space. These patterns are essential for comprehending how consumers make decisions. Practically speaking, XGWO’s high precision and recall indicate it can correctly categorize customer behavior while minimizing false positives and negatives. In e-commerce contexts, where precise forecasting of consumer behavior directly influences inventory control, customer retention tactics, and tailored marketing, this equilibrium is crucial. When applied to fresh, untested consumer data, the model’s strong generalization ability, as demonstrated by robust test metrics, further validates its reliability.

As a result, the hybrid XGWO model is a breakthrough in the study of consumer behavior in e-commerce. Its improved stability and predictive accuracy provide insights that help businesses make more focused, efficient decisions. In order to gain a deeper understanding of complex consumer datasets and enhance the overall e-commerce experience, it is crucial to combine cutting-edge ML models with sophisticated optimization techniques.

**Table 3:** Evaluation metrics were employed to measure the predictive accuracy and overall performance of the models.

Phase	Category	Models	Metrics			
			Accuracy	Precision	Recall	F1 Score
Train	Single	XGB	0.960	0.961	0.960	0.960
		LGB	0.950	0.952	0.950	0.950
		CAT	0.929	0.930	0.929	0.929
	Hybrid	XGWD	0.976	0.976	0.976	0.976
		XGWO	0.989	0.989	0.989	0.989
		LGWD	0.967	0.968	0.967	0.967
		LGWO	0.981	0.982	0.981	0.982
		CAWD	0.949	0.950	0.949	0.949
		CAWO	0.964	0.965	0.964	0.964
Test	Single	XGB	0.943	0.943	0.943	0.943
		LGB	0.933	0.934	0.933	0.933
		CAT	0.917	0.919	0.917	0.917
	Hybrid	XGWD	0.960	0.960	0.960	0.960
		XGWO	0.973	0.973	0.973	0.973
		LGWD	0.953	0.954	0.953	0.953
		LGWO	0.967	0.967	0.966	0.966
		CAWD	0.930	0.930	0.930	0.930
		CAWO	0.947	0.947	0.947	0.947

**Fig. 8:**
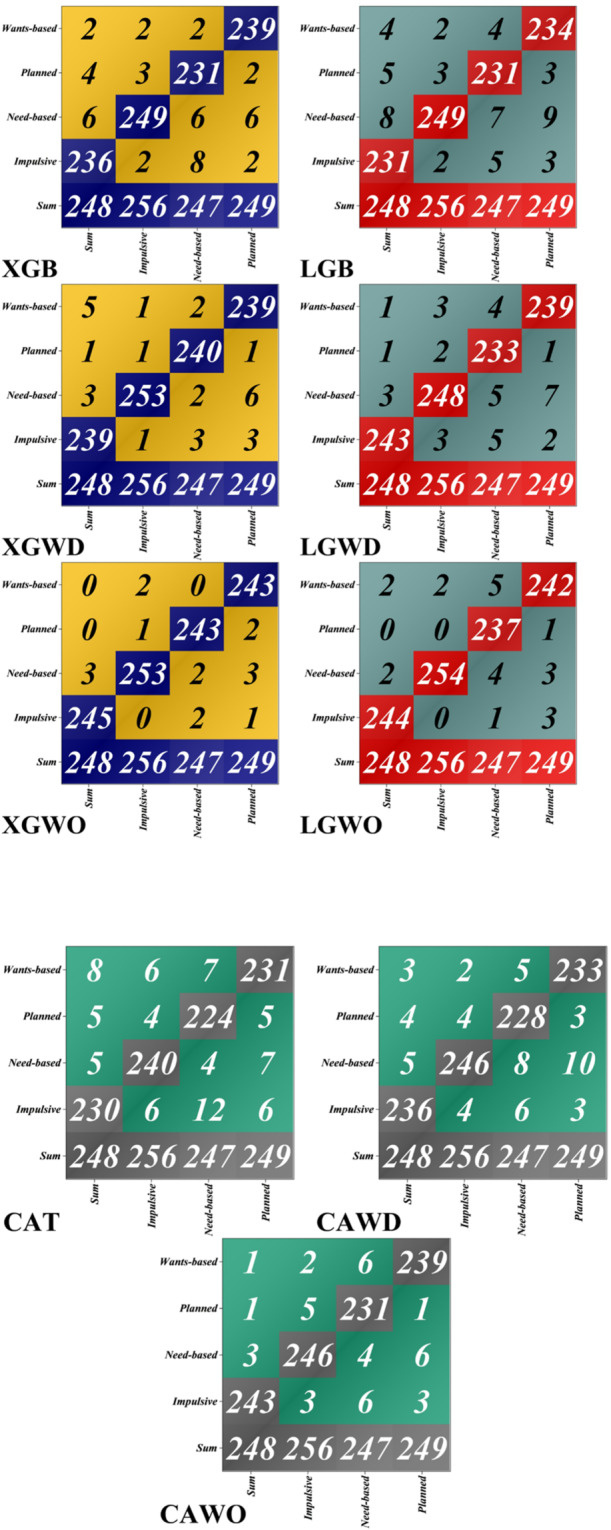
Confusion matrix for evaluating model accuracy under different conditions.

Figure 8’s confusion matrix analysis shows that hybrid models tuned with bio-inspired optimization perform noticeably better than their default counterparts in classifying four important consumer segments: impulsive, need-based, planned, and wants-based purchases. At first, the baseline XGBoost and LightGBM models did well; XGBoost was especially good at detecting need-based purchases. Untuned hyperparameters were suboptimal for this multi-class e-commerce scenario, as LightGBM’s default settings produced lower accuracy for wants-based and impulsive segments. Significant performance gains were obtained when the WDOA was applied to both XGBoost and LightGBM. Compared to the base XGBoost, XGBoost with WDOA (XGWD) improved the number of accurate predictions and reduced misclassifications in the planned, need-based, and impulsive categories. Targeted hyperparameter tuning is beneficial for even powerful classifiers, as demonstrated by LightGBM with WDOA (LGWD), which showed notable gains, particularly in the impulsive segment. These improvements highlight WDOA’s capacity to extract complex patterns of consumer behavior from high-dimensional e-commerce data. Using the WaOA yielded additional benefits. Across all four consumer categories, the XGBoost-WaOA hybrid (XGWO) achieved the lowest misclassification counts and the highest correct-classification rates, making it the best model. To illustrate its superior ability to interpret complex consumer signals, XGWO demonstrated near-perfect discrimination among wants-based, planned, need-based, and impulsive purchases. Comparably good results were obtained by LightGBM‐WaOA (LGWO), which at times outperformed XGWO in need-based classifications. This suggests that LightGBM’s tree structures can capture behavioral subtleties with comparable effectiveness when adjusted. With higher misclassification rates and lower overall accuracy, CatBoost’s default version lagged behind XGBoost and LightGBM. Nonetheless, CatBoost-WaOA (CAWO) and CatBoost-WDOA (CAWD) both demonstrated notable gains, demonstrating that bio-inspired optimizers successfully improve CatBoost’s management of categorical variables. Despite these improvements, optimized CatBoost versions did not outperform XGWO or LGWO, suggesting that, when properly adjusted, XGBoost and LightGBM architectures perform better with this dataset. After optimization, precision and recall metrics significantly improved for all models. Notably, the most important factors influencing classification accuracy were Product Rating, Customer Satisfaction, and Customer Loyalty Program Membership. These variables directly enhance the models’ discriminatory power by capturing fundamental aspects of consumer sentiment and engagement.

In conclusion, hyperparameter optimization, whether through WaOA or WDOA, proved crucial to attaining high-fidelity classification of consumer behavior in e-commerce. The XGWO model demonstrated the best performance, differentiating among a variety of purchasing motivations in real time. Businesses can apply more precise customer targeting, customize promotions for specific consumer segments, and improve inventory management with the help of such sophisticated classification capabilities. These results thus confirm that combining gradient boosting frameworks with bio-inspired optimization algorithms is a powerful method for extracting useful information from intricate e-commerce datasets.

A thorough analysis of several ML models for categorizing e-commerce consumer behaviors is shown in Table 4, which is divided into four main groups: impulsive, need-based, planned, and wants-based buying patterns. Interestingly, the hybrid models outperform their single-model counterparts, with the XGWO hybrid model showing the best performance in every behavioral condition. The XGWO model’s remarkable ability to correctly identify patterns in consumer behavior is demonstrated by its consistently high precision, recall, and F1-score metrics, which range from 0.9798 to 0.9879. In the Impulsive and Planned categories, where F1-scores can reach 0.9879 and 0.9858, respectively, this level of performance is particularly noticeable. In consumer behavior analysis, it is crucial to minimize false positives and false negatives, and these metrics indicate a balanced, dependable model performance. Accurate classification models like XGWO are essential when discussing consumer behavior in e-commerce. E-commerce platforms can create precisely calibrated segmentation strategies by identifying subtle behavioral patterns. For example, recognizing need-based and planned buyers enables the delivery of tailored recommendations aligned with customer intent and purchase cycles, while identifying impulsive buyers enables targeted promotional campaigns to capitalize on impulsive purchasing tendencies.

Furthermore, the XGWO model’s better performance highlights the effectiveness of gradient boosting algorithms when combined with sophisticated optimization strategies to improve prediction robustness and generalization. By successfully capturing intricate, non-linear relationships within e-commerce transaction data, this hybrid approach enables a deeper understanding of consumer decision-making processes. The use of such sophisticated hybrid models ultimately advances the field of e-commerce consumer behavior by increasing customer engagement and retention, refining marketing strategies, and improving the accuracy of consumer segmentation. The results confirm that hybrid modeling frameworks, such as the XGWO model, are a useful methodological development for e-commerce predictive analytics.

**Table 4:** Models’ performance under various conditions.

Category	Model	Condition	Metric		
			Precision	Recall	F1-Score
Single	XGB	Impulsive	0.9516	0.9516	0.9516
		Need-based	0.9326	0.9727	0.9522
		Planned	0.9625	0.9325	0.9487
		Wants-based	0.9755	0.9598	0.9676
	LGB	Impulsive	0.9585	0.9315	0.9448
		Need-based	0.9121	0.9727	0.9414
		Planned	0.9545	0.9352	0.9448
		Wants-based	0.959	0.9398	0.9493
	CAT	Impulsive	0.9055	0.9274	0.9163
		Need-based	0.9375	0.9375	0.9375
		Planned	0.9412	0.9069	0.9237
		Wants-based	0.9167	0.9277	0.9222
Hybrid	XGWD	Impulsive	0.9715	0.9637	0.9676
		Need-based	0.9583	0.9883	0.9731
		Planned	0.9877	0.9717	0.9796
		Wants-based	0.9676	0.9598	0.9637
	XGWO	Impulsive	0.9879	0.9879	0.9879
		Need-based	0.9693	0.9883	0.9798
		Planned	0.9878	0.9838	0.9858
		Wants-based	0.9918	0.9759	0.9838
	LGWD	Impulsive	0.9605	0.9798	0.9701
		Need-based	0.943	0.9688	0.9557
		Planned	0.9831	0.9433	9628
		Wants-based	0.9676	0.9598	0.9637
	LGWO	Impulsive	0.9839	0.9839	0.9839
		Need-based	0.9658	0.9922	0.9788
		Planned	0.9958	0.9595	0.9773
		Wants-based	0.9641	0.9719	0.968
	CAWD	Impulsive	0.9478	0.9516	0.9497
		Need-based	0.9145	0.9609	0.9371
		Planned	0.954	0.9231	0.9383
		Wants-based	0.9588	0.9357	0.9472
	CAWO	Impulsive	0.9529	0.9798	0.9662
		Need-based	0.9498	0.9606	0.9559
		Planned	0.9706	0.9352	0.9526
		Wants-based	0.9637	0.9598	0.9618

**Fig. 9:**
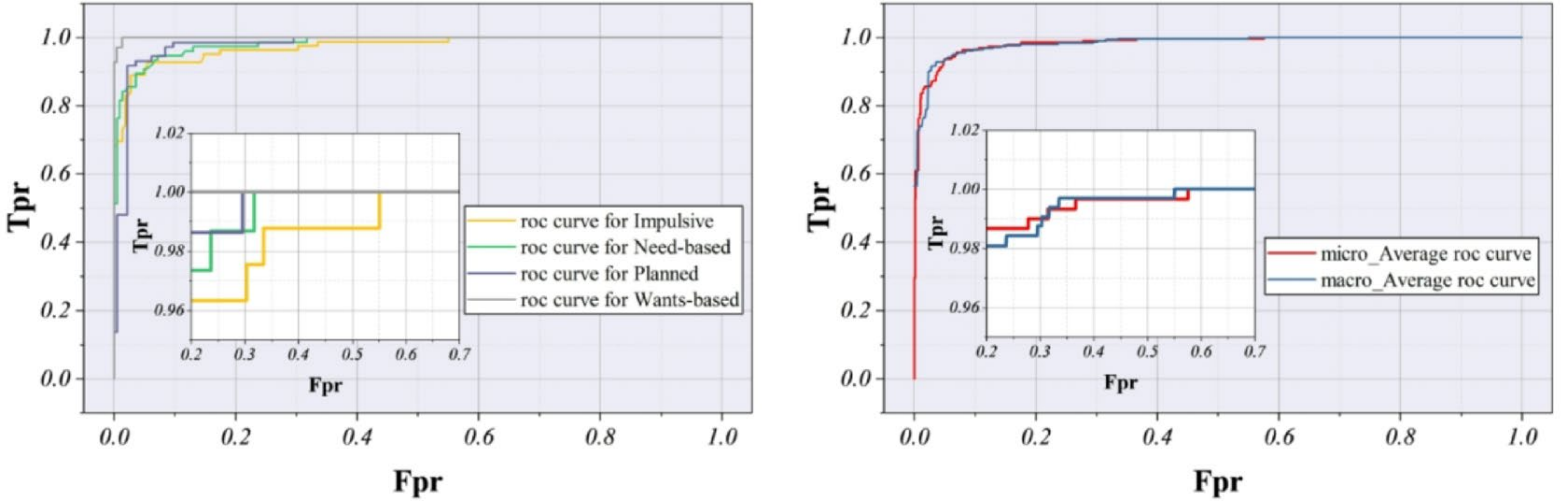
The ROC curve for the models’ ability to distinguish between classes emphasizes their sensitivity and specificity across different thresholds.

The hybrid classification model, especially the XGWO-enhanced variant, performs well in accurately predicting consumer behavior based on the e-commerce consumer behavior, according to the ROC curve analysis in Fig. 9. Excellent sensitivity and specificity are indicated by the ROC curves, which show a high True Positive Rate and a low False Positive Rate for all classes. The ideal path closely follows the macro-average and micro-average curves, indicating dependable, consistent model behavior. With ROC curves for Need-based and Wants-based consumer behaviors almost matching the average curves and indicating little variance, the model notably demonstrates remarkable accuracy in identifying these consumer behaviors. The overall performance remains strong, even though the Impulsive and Planned categories exhibit modest decreases in sensitivity at specific thresholds. Strong discriminatory power is confirmed by visual cues that indicate AUC values for all classes are greater than 0.98. These findings support the model’s applicability to targeted marketing, tailored recommendations, and the development of a more comprehensive e-commerce strategy by demonstrating how well it differentiates across consumer decision models.

To verify whether the observed performance differences among models were statistically significant, a Wilcoxon signed-rank test was conducted on the cross-validation results. As shown in Table 5, all pairwise comparisons yielded p-values greater than 0.05, indicating that the performance differences among the evaluated models are not statistically significant at the 95% confidence level. Although certain hybrid configurations (e.g., XGWO and LGWD) achieved higher mean metric values, the Wilcoxon analysis suggests that these improvements should be interpreted as performance tendencies rather than statistically confirmed superiority. This statistical validation ensures that the reported ranking of models is supported by non-parametric significance testing rather than relying solely on metric magnitude.

**Table 5:** Result of the Wilcoxon analyses for the developed models.

Model	Stat	p_value	Rank_p	Rank_stat
XGWO	42	0.1685	5	1
XGWD	192.5	0.5836	1	3
XGB	397	0.1638	6	6
LGWO	118.5	0.5451	3	2
LGWD	227.5	0.0553	9	4
LGB	684	0.4615	4	8
CAWO	290.5	0.0628	8	5
CAWD	616.5	0.0875	7	7
CAT	1315.5	0.5562	2	9

**Fig. 10:**
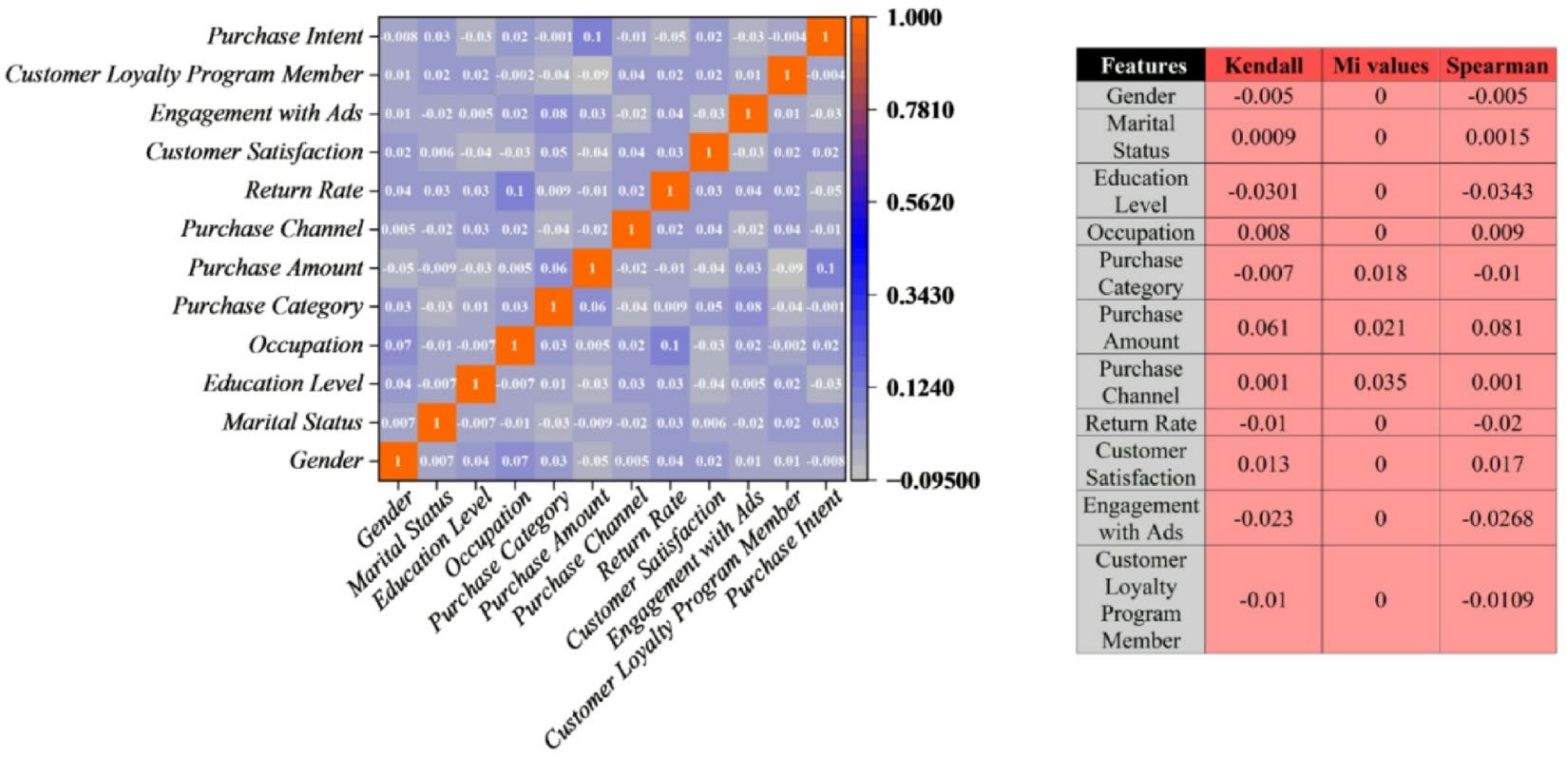
The correlation plot after feature selection visualizes the copula-based dependencies among the selected input features, highlighting their joint distribution and interrelationships.

In the context of e-commerce consumer behavior, the most prominent feature influencing Purchase Intent is Customer Loyalty Program Membership, according to the copula-based dependency analysis using Kendall’s Tau. Since Kendall’s Tau in Fig. 10 provides a strong non-parametric evaluation of monotonic relationships in contrast to traditional linear correlation measures, it is especially well-suited for the mixed-type data found in e-commerce datasets, which frequently contain numerical, ordinal, and categorical variables. Customers who participate in loyalty programs have a significantly higher propensity to purchase, according to the analysis, which shows a strong, positive, monotonic association between loyalty program membership and purchase intent. This outcome aligns with accepted theoretical frameworks in consumer behavior, which emphasize the importance of loyalty programs in retaining customers and increasing their likelihood of purchasing in online retail environments.

While there is a positive correlation between Purchase Intent and other features like Purchase Amount and Customer Satisfaction, their respective strengths are relatively moderate. The strongest predictor of purchase intent is loyalty program participation, as evidenced by the weak or nonexistent monotonic correlations between demographic factors and other behavioral variables, including engagement with advertisements, return rate, and purchase channel. These results highlight the strategic significance of customer loyalty programs in influencing consumer behavior in e-commerce. This implies that increasing loyalty program participation should be a top priority for both researchers and practitioners to influence purchase intentions effectively. In conclusion, Customer Loyalty Program Membership stands out as the most effective feature for modeling and comprehending purchase intent within the e-commerce consumer behavior framework. This provides crucial insights for focused marketing campaigns and customer retention initiatives in digital commerce environments.

## Discussion

The results of this study highlighted the advantages of hybrid modeling frameworks in analyzing e-commerce consumer behavior, particularly the XGWO-enhanced classifier. The model demonstrated near-perfect discrimination between consumer behavior classes, achieving high true positive rates with low false positive rates. Need-based and Wants-based segments exhibited especially strong performance, indicating that the model effectively distinguished between hedonic and utilitarian motivations, which was critical for demand forecasting and targeted marketing. Overall AUC values exceeded 0.98, confirming reliable classification even for complex behavioral categories. Class-specific metrics further supported the XGWO model’s superiority. F1-scores ranged from 0.9798 (Need-based) to 0.9879 (Impulsive), reflecting a robust balance between precision and recall. High recall for Need-based and Planned segments ensured accurate identification of intentional or necessity-driven purchases, facilitating personalized recommendations and inventory optimization.

Additionally, the model’s ability to identify impulsive buyers enabled timely marketing interventions, such as limited-time offers. The incorporation of the WaOA for hyperparameter tuning contributed to these performance gains. XGWO converged faster than other hybrids, achieving an accuracy of approximately 0.98, which supported frequent model retraining in high-velocity e-commerce settings. By balancing regularization and complexity, XGWO avoided overfitting while capturing multifaceted feature relationships, including Product Rating, Customer Satisfaction, and Loyalty Program membership, which were identified as key predictors of purchase intent. Compared to other gradient boosting hybrids (XGB, LGB, CAT-based), XGWO delivered superior sensitivity, specificity, and convergence speed, enabling practical applications such as precise customer targeting, dynamic pricing, improved marketing ROI, and proactive retention strategies. Feature importance rankings enhanced interpretability and adherence to data governance standards. Overall, the XGWO hybrid model provided a scalable, effective framework for deriving actionable insights from complex consumer datasets, offering both methodological rigor and operational utility in e-commerce predictive analytics.

### Practical impacts and strategic advantages of advanced consumer behavior modeling

Advanced consumer behavior modeling offers substantial strategic value for digital retailers by enabling sharper customer targeting and personalization. Accurate real-time prediction of purchase intentions allows organizations to deliver highly relevant product recommendations, promotional offers, and content, thereby streamlining the customer journey and improving engagement metrics such as click-through and conversion rates. Enhanced predictive precision also contributes to stronger brand loyalty by aligning marketing communication with individual behavioral patterns. In parallel, optimized hyperparameter tuning through WaOA and WDOA allows models to extract maximal information from available data, improving campaign efficiency and reducing waste in advertising expenditure. More accurate audience targeting leads directly to improved marketing return on investment and more efficient budget allocation.

Beyond marketing performance, the proposed framework supports operational decision-making through dynamic pricing and inventory optimization. Reliable behavioral forecasts enable near real-time adjustment of pricing strategies and stock levels in response to demand fluctuations, competitor actions, and emerging trends. This adaptability reduces markdown losses, minimizes stockouts, and increases revenue per visitor. Furthermore, the ability to detect subtle behavioral shifts facilitates proactive churn prevention through automated and personalized retention strategies, such as targeted incentives or customized support interventions. These predictive retention mechanisms are typically more cost-effective than reacquisition strategies and contribute to long-term customer value.

From a strategic perspective, the integration of fast-convergent bio-inspired optimization algorithms enhances operational scalability and organizational agility. Frequent model retraining on evolving data streams becomes computationally feasible, allowing businesses to refine campaigns and product strategies based on current consumer insights rather than static historical reports. The incorporation of psychologically and behaviorally grounded features also improves interpretability, supporting transparent and accountable decision-making in compliance with data protection regulations. Finally, early adoption of optimization-enhanced hybrid modeling frameworks provides competitive differentiation, enabling organizations to manage high-dimensional, high-velocity data more effectively than competitors relying on static or manually tuned systems.

### Limitations and potential sources of bias

Despite the strong predictive performance achieved by the proposed hybrid framework, several limitations should be acknowledged.

First, the dataset was obtained from a publicly available Kaggle repository. Although it contains rich behavioral and transactional variables, its representativeness of real-world e-commerce ecosystems may be limited. The data may reflect specific demographic distributions or simulated patterns that do not fully capture platform-specific dynamics, seasonal variations, or cross-cultural purchasing differences. Consequently, external validity across different retail platforms and geographic regions should be interpreted cautiously.

Second, the classification of purchase intent relies on labeled categories that may not fully reflect the psychological complexity of consumer decision-making. Constructs such as impulsive or planned behavior are inherently multifaceted and context-dependent. The operationalization of these categories in structured datasets may oversimplify underlying cognitive and emotional processes, potentially introducing construct validity bias.

Third, although the dataset did not exhibit significant class imbalance or missing values, other forms of bias may remain. For example, self-reported satisfaction scores, loyalty indicators, and product ratings may be subject to response bias or social desirability effects. Similarly, engagement metrics may reflect platform-specific algorithmic exposure rather than purely consumer-driven behavior.

Fourth, while integrating metaheuristic optimization improves hyperparameter search efficiency, it also increases computational complexity. The scalability of such hybrid frameworks in extremely large-scale, real-time production environments requires further empirical validation.

Finally, although stratified splitting was employed, the evaluation was conducted within a single dataset environment. Cross-platform validation (e.g., training on historical data and testing on future periods) was not conducted, which limits conclusions about the long term.

Future research should therefore explore multi-platform datasets, incorporate longitudinal validation strategies, and investigate causal modeling approaches to complement predictive analysis.

### Pros and cons of study

This study presents several methodological and practical strengths.

- First, it introduces a structured hybrid optimization framework that integrates two recent bio-inspired metaheuristic algorithms (WaOA and WDOA) with three state-of-the-art gradient-boosting classifiers within a unified evaluation setting. This systematic comparison provides clearer methodological guidance than studies relying on single-model implementations.
- Second, the research explicitly distinguishes between impulsive and planned (need-based) purchase behavior, offering a psychologically grounded classification approach rather than modeling generic purchase intention.
- Third, the study incorporates multidimensional consumer features, including demographic, transactional, engagement, and behavioral variables, enhancing model richness and interpretability.
- Fourth, the evaluation strategy includes multiple performance metrics and class-specific F1-scores, ensuring robust performance assessment rather than relying solely on overall accuracy.
- Finally, integrating feature importance analysis enhances interpretability and managerial relevance, thereby supporting data-driven decision-making in e-commerce contexts.

Despite these strengths, several limitations should be acknowledged.

- First, the dataset was obtained from a publicly available source, which may limit external validity across different e-commerce platforms and geographic contexts.
- Second, although metaheuristic optimization improves hyperparameter tuning, it increases computational cost compared to conventional search strategies, potentially affecting scalability in large-scale production environments.
- Third, the study focuses on predictive modeling rather than causal inference; therefore, feature importance reflects statistical association rather than deterministic behavioral drivers.
- Fourth, evaluation was conducted within a single dataset environment without cross-platform or temporal validation, which may constrain generalizability under dynamic market conditions.

## Conclusion

This study examined multiple factors influencing e-commerce consumer behavior, including demographics, purchase metrics, product feedback, and decision-making indicators, enabling the distinction of impulsive, need-based, planned, and want-based transactions. By leveraging such multidimensional data, the research advances both theoretical understanding and practical applications in consumer segmentation and predictive analytics. A primary contribution of this work is the development of a hybrid optimization framework combining bio-inspired metaheuristic algorithms—WaOA and WDOA—with gradient-boosting classifiers (CatBoost, XGBoost, and LightGBM). Unlike traditional hyperparameter tuning methods, these algorithms achieve faster convergence toward near-optimal configurations by effectively balancing exploration and exploitation during training. Experimental results demonstrated that the XGBoost classifier optimized via WaOA (XGWO) outperformed all single-model baselines and other hybrid configurations. XGWO achieved F1-scores of 0.9879 for impulsive purchases and 0.9798 for need-based purchases, with training and testing accuracy of 0.989 and 0.973, respectively. Feature importance analysis identified Product Rating, Customer Satisfaction, and Loyalty Program Membership as critical predictors of purchase intent. ROC analysis confirmed XGWO’s strong multi-class discrimination, with low false-positive rates, high true-positive rates, and overall AUC values exceeding 0.98. The model consistently distinguished between behavioral classes, particularly need- and wants-based segments, with minimal variance. These findings have multiple practical implications. E-commerce platforms can enhance customer targeting, personalization, and retention by accurately forecasting purchase intentions. Hyperparameter optimization reduces unnecessary advertising expenditure, improving marketing ROI. Accurate real-time predictions support dynamic pricing, inventory optimization, and proactive churn prevention. The framework’s rapid convergence allows frequent retraining on updated datasets with minimal computational cost, ensuring operational scalability. Furthermore, incorporating cultural and psychological factors enhances interpretability and compliance with data governance standards. Generally, the XGWO hybrid model represents a methodological advance in e-commerce analytics, combining predictive accuracy, efficiency, and transparency. It provides a robust foundation for both scholarly research and practical applications, enabling data-driven strategies that maximize customer engagement, operational effectiveness, and strategic decision-making. Despite these contributions, several avenues for future research remain. First, validation across multiple real-world e-commerce datasets is necessary to assess the generalizability of the proposed framework across platforms, regions, and cultural contexts. Second, longitudinal evaluation using time-based splits would provide insight into model stability under evolving consumer trends and seasonal demand fluctuations. Third, future studies should explore real-time deployment challenges, including computational scalability, latency constraints, and integration with live recommendation or pricing systems. Fourth, incorporating causal modeling techniques or experimental validation could help distinguish correlation from underlying behavioral mechanisms.

## Data Availability

The dataset and codes used in this study is publicly available on https://github.com/Nacui442/Explaining-and-Predicting-Consumer-Decision-Making-in-E-Commerce.
